# Non-commutativity and Local Indistinguishability of Quantum States

**DOI:** 10.1038/srep06336

**Published:** 2014-09-11

**Authors:** Teng Ma, Ming-Jing Zhao, Yao-Kun Wang, Shao-Ming Fei

**Affiliations:** 1 School of Mathematical Sciences, Capital Normal University, Beijing 100048, P. R. China; 2 Department of Mathematics, School of Science, Beijing Information Science and Technology University, Beijing 100192, P. R. China; 3 Institute of Physics, Chinese Academy of Sciences, Beijing 100190, P. R. China; 4 College of Mathematics, Tonghua Normal University, Tonghua 134001, P. R. China; 5 Max-Planck-Institute for Mathematics in the Sciences, Leipzig 04103, Germany

## Abstract

We study the local indistinguishability problem of quantum states. By introducing an easily calculated quantity, non-commutativity, we present an criterion which is both necessary and sufficient for the local indistinguishability of a complete set of pure orthogonal product states. A constructive distinguishing procedure to obtain the concrete local measurements and classical communications is given. The non-commutativity of ensembles can be also used to characterize the quantumness for classical-quantum or quantum-classical correlated states.

Nonlocality is one of the most important features in quantum mechanics. Quantum entanglement was firstly introduced to characterize and quantify the nonlocality, as it acts as a crucial resource in many quantum information processing tasks^1^. On the other hand, there are also quantum information and computation processing tasks without quantum entanglement, like the quantum computation with one qubit (DQC1)^2^. Other types of quantum correlations such as quantum discord^3^ are introduced to capture this kind of quantum advantage. The quantum discord is considered more general than entanglement and captures the quantum correlations that entanglement fails to capture^4^.

A well known quantum phenomenon that exhibits quantum nonlocality is the local indistinguishability for some quantum states. State discrimination or distinguishing is essentially primitive for many quantum information tasks, such as quantum cryptography^5^ and quantum algorithms^6^. Moreover, with the remarkable experimental advances in preparation and measurement of quantum states^7^,^8^, it becomes essential to have a theory to assess the performance of quantum state discrimination protocols. One basic problem of state discrimination is judging the local distinguishability for a set of pure orthogonal product states (POPS). Consider a set of bipartite POPS, each with a prior probability, which constitutes an ensemble of a density operator *ρ_AB_*. Although a set of POPS can always be distinguished globally, it may not be distinguished locally by local operations and classical communications (LOCC). This is called *quantum nonlocality without entanglement*^9^. In^10^ the relation between quantum discord and local indistinguishability has been investigated. However, contrary to one's intuitive appeal, the quantum discord of *ρ_AB_* is not an indicator of local indistinguishability for a set of states that constitute the pure-state decomposition of *ρ_AB_*^4^,^10^. It shows no relation between zero quantum correlation and the local distinguishability for a set of POPS. The local indistinguishability problem of a set of POPS remains open, although many research have been done so far^9^,^10^,^11^,^12^,^13^,^14^,^18^.

It is natural to ask what kind of quantity or quantumness accounts for the quantum nonlocality. In this article, instead of quantum correlation measures, such as quantum discord, quantum deficit, etc., we investigate local indistinguishability for a set of POPS from the point of quantumness of ensemble. We first introduce an easily calculated quantity, non-commutativity, to quantify the quantumness of a quantum ensemble. Based on the non-commutativity, we present a necessary and sufficient criterion for the local indistinguishability. We also give a constructive distinguishing procedure to judge the local indistinguishability for any given set of POPS by using the criterion. Moreover, by proving the uniqueness of the expression of for semi-classical quantum correlated states, we show that our definition for quantumness of ensembles can be used to characterize the quantumness for semi-classical states.

## Results

### Non-commutativity for a set quantum states

A quantum ensemble containing only two pure states |*ψ*〉 and |*ϕ*〉 with equal probability can be viewed as a set of binary signals in some communication scheme. If |*ψ*〉 and |*ϕ*〉 are orthogonal, the ensemble becomes classical since different states of classical information can be thought of merely as orthogonal quantum states^1^. Non complete overlap of the two states, *x* = |〈*ψ*|*ϕ*〉|, 0 < *x* < 1, induces quantumness for the ensemble. In fact the ensemble is most ‘quantum’ when 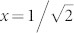, i.e. when the two states have an angle 45° between them^15^.

Taking the above observation into account, we introduce non-commutativity to characterize the quantumness for a quantum ensemble. Let *A*_1_, *A*_2_, …, *A_n_* be a set of operators. We define the total non-commutativity for this set, 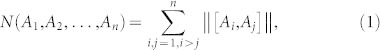
where [*A*, *B*] = *AB* − *BA*, ||*A*|| is the trace norm of the operator *A*, 
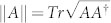
. The non-commutativity is a natural measure of ‘quantumness’ for a quantum ensemble *ε* = {*p_i_*, *ρ_i_*}, where *ρ_i_* are density operators with probability *p_i_*. Denote *A_i_* = *p_i_ρ_i_*, then *N*(*A*_1_, *A*_2_, …, *A_n_*) is the measure of quantumness for the ensemble *ε*. Here for the problem of local distinguishability of states *ρ*_1_, *ρ*_2_, …, *ρ_n_*, the prior probabilities *p_i_* are irrelevant. One only needs to concern the quantity *N*(*ε*) = *N*(*ρ*_1_, *ρ*_2_, …, *ρ_n_*) to judge the local distinguishability of the set of states *ρ*_1_, *ρ*_2_, …, *ρ_n_*. The prior probabilities *p_i_* do make sense when one concerns the quantumness of a state given by the ensemble *ε* = {*p_i_*, *ρ_i_*} (see section “The quantumness of semi-classical states”).

The non-commutativity *N* has the following properties, which make it a well defined measure for quantumness of an ensemble:

1. *N* is non negative;
2. *N* is unitary invariant, *N*(*ε*) = *N*(*UεU*^†^), where *UεU*^†^ = {*p_i_*, *Uρ_i_U*^†^} with *U* being any unitary matrix;
3. For an ensemble only containing two pure states *A*_1_ = |*ψ*〉 〈*ψ*| and *A*_2_ = |*ϕ*〉 〈*ϕ*|, without considering their prior probabilities, *N*(*A*_1_, *A*_2_) is zero only when the overlap *x* = |〈*ϕ*|*ψ*〉| = 0 or 1. *N*(*A*_1_, *A*_2_) gets to a maximum of 1 when 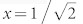 (see Methods for the proof), which coincides with the above analysis of quantumness for two pure states;
4. The sum of two sets' non-commutativity is equal to or less than the non-commutativity of the sum of the two sets: *N*({|*a_i_*〉}) + *N*({|*b_i_*〉}) ≤ *N*({{|*a_i_*〉}, {|*b_i_*〉}}). One can easily verify the inequality from the definition of *N*. The equality holds if {|*a_i_*〉} and {|*b_i_*〉} are either mutually orthogonal or identical.

A set of quantum states corresponds to a quantum ensemble. Nevertheless, one density operator may have many quantum ensemble decompositions. For instance, consider the density operator in a 3 ⊗ 3 system, 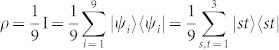, where |*ψ*_1_〉 = |1〉|1〉, 
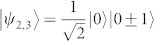
, 
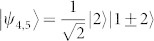
, 
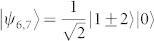
, 
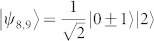
, |*st*〉 = |*s*〉 ⊗ |*t*〉, |*s*〉 and |*t*〉 are the computational basis. Both {|*ψ_i_*〉} and {|*st*〉} are pure-state decompositions of *ρ*. However, the set {|*st*〉} can be locally distinguished while {|*ψ_i_*〉} cannot^9^. Hence that *ρ* having zero quantum correlation is not an indicator for the local distinguishability of the states in *ρ*'s pure-state decomposition. On the other hand, if we change the probability of |*ψ_i_*〉, *ρ* is no longer an identity and can have nonzero quantum correlation, but the nine states |*ψ_i_*〉 still remain locally indistinguishable. Usually, the local indistinguishability of a set of pure states has no simple relations with the properties of the related density operator^4^.

To study the local indistinguishability, and its relations with the quantumness of a quantum ensemble in terms of non-commutativity, in the following we call an ensemble *ε* = {*p_i_*, |*ψ_i_*〉} classical if *N*(*ε*) = 0, and quantum if *N*(*ε*) > 0. If *N*(*ε*) = 0, from the properties of non-commutativity, {|*ψ_i_*〉} must be either mutually orthogonal or identical and the states form a set of classical signals from informatics point of view. If *N*(*ε*) > 0, among {|*ψ_i_*〉} there must be at least one pair of states that are neither orthogonal nor identical. Consider a set of bipartite POPS {|*ψ_i_*〉 = |*a_i_*〉 ⊗ |*b_i_*〉}, where |*ψ_i_*〉 are all mutually orthogonal, |*a_i_*〉 and |*b_i_*〉 are associated to partite *A* and *B* respectively. Obviously, this set forms a classical ensemble *ε* = {*p_i_*, |*ψ_i_*〉} since *N*(*ε*) = 0, where *p_i_* is the probability with respect to |*ψ_i_*〉. Correspondingly one has ensembles *ε^A^* = {*p_i_*, |*a_i_*〉} and *ε^B^* = {*p_i_*, |*b_i_*〉} respectively. If *N*(*ε^A^*) = *N*(*ε^B^*) = 0, we call *ε* a classical-classical ensemble. If *N*(*ε^A^*) = 0, *N*(*ε^B^*) > 0, we call *ε* a classical-quantum ensemble. Analogously we can define quantum-classical and quantum-quantum ensembles.

### Local distinguishability for a set quantum states

A set of states is said to be reliably distinguished locally or distinguished locally if all the states in the set can be distinguished by *finite* rounds of LOCC protocols. If there are at least two states in a set which can not be distinguished by finite rounds of LOCC protocols, we say that the set can not be distinguished locally. The arguably more operational “asymptotic local operations and classical communications discrimination problem^16^,^17^” is not under our consideration.

**Theorem 1** If a set of bipartite pure orthogonal product states cannot be locally distinguished, the ensemble composed of these states must be a quantum-quantum ensemble.

**Proof**. Consider a set of bipartite POPS, not necessarily complete, {|*ψ_i_*〉 = |*a_i_*〉 ⊗ |*b_i_*〉} with 〈*ψ_i_*|*ψ_j_*〉 = 0, ∀*i* ≠ *j*. Suppose these states form a classical-quantum or classical-classical ensemble *ε* = {*p_i_*, |*a_i_*〉 ⊗ |*b_i_*〉} with non zero *p_i_*. Since *N*({|*a_i_*〉}) = 0, {|*a_i_*〉} must be either mutually orthogonal or identical. To distinguish {|*ψ_i_*〉} locally, we can first take projective measurement on *A* side to distinguish those orthogonal states in {|*a_i_*〉}. For those identical states |*a_i_*〉, for example, |*a*_1_〉 = |*a*_2_〉, we can take a projective measurement on *B* to distinguish state |*a*_1_〉 from state |*a*_2_〉, since |*b*_1_〉 and |*b*_2_〉 must be mutually orthogonal to ensure that 〈*ψ*_1_|*ψ*_2_〉 = 0. In this way we can distinguish the states in the set *ε* reliably. The analysis is similar if *ε* is a quantum-classical ensemble. Therefore if a set of bipartite POPS forms a classical-classical, quantum-classical or classical-quantum ensemble, the set can be locally distinguished. And if the set of POPS states cannot be locally distinguished, the ensemble composed of these states must be a quantum-quantum one. □

Since it is impossible to form a quantum-quantum ensemble for a set of POPS in a 2 ⊗ 2 system, Theorem 1 is both necessary and sufficient for 2 ⊗ 2 systems. For higher dimensional cases, there are quantum-quantum ensembles whose states can be locally distinguished. In fact, for 2 ⊗ *n* systems, any set of POPS is locally distinguishable^11^. Theorem 1 reveals a relation between local distinguishability and quantumness of a set of states. In the following, we give a necessary and sufficient criterion for local distinguishability for all POPS from the view of quantumness of ensembles.

We say that two sets {|*a_i_*〉} and {|*a*′*_j_*〉} are orthogonal if and only if 〈*a_i_*|*a*′*_j_*〉 = 0, ∀*i*, *j*. If a set of states cannot be divided into subsets such that those subsets are mutually orthogonal, then we call the set a single set. Consider a set of states *ε* = {|*a*_1_〉 = |0〉, |*a*_2_〉 = |0 + 1〉, |*a*_3_〉 = |2〉}. This set can be divided into two single subsets *ε*_1_ = {|*a*_1_〉, |*a*_2_〉} and *ε*_2_ = {|*a*_3_〉} which are orthogonal and each subset cannot be split further. We call this partition the direct sum decomposition and denote it as *ε* = *ε*_1_ ⊕ *ε*_2_. A single set means that the set cannot be decomposed into such direct sums. If all the subsets are single sets in a direct sum decomposition, we say that the decomposition is a single set decomposition. It can verified that the single set decomposition of a non-single set is unique. For a single set, adding some states in the vector space spanned by itself will keep the set a single one. Nevertheless, taking away some states from a single set, the set could become a non-single one. In the following, by a set of states' decomposition we mean the single set decomposition.

**Lemma** For a set of states *ε* = {|*a_i_*〉}, *m* = dim(span{|*a_i_*〉}), the following statements are equivalent: (a) *ε* is a single set. (b) There are *m* linear independent states 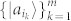 in *ε* satisfying the following relations: 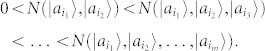
(2)(c) A nondestructive projective measurement, a measurement which keeps the quantum state unchanged^18^, can do nothing to distinguish the states in *ε*.

See Methods for the proof of the Lemma. From the Lemma we have

**Theorem 2** For a complete set of *m* ⊗ *n* POPS, *ε* = {|*ψ_i_*〉 = |*a_i_*〉 ⊗ |*b_i_*〉} with 〈*ψ_i_*|*ψ_j_*〉 = 0, ∀*i* ≠ *j*, the set *ε* cannot be completely locally distinguished if and only if there exist subsets 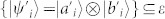, such that 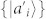 and 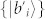 are all single sets, i.e., there exist 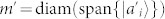 linear independent 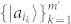 in 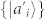 and 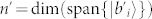 linear independent 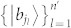 in 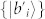 satisfying 
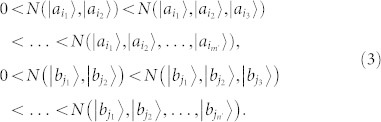
**Proof**. Recall that a complete set of POPS can be locally distinguished if and only if the states can be distinguished by local nondestructive projective measurement and classical communication^18^. If a set of states in *ε* cannot be completely locally distinguished by local nondestructive projective measurement and classical communication, there must exist a subset 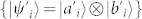 of *ε* such that 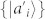 and 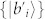 are all single sets (Lemma 1). Obviously, the converse is also right. From Lemma 1 we have that the formula (3) holds if and only if 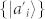 and 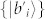 are single sets. □

From the view of accessible information, the more quantum an ensemble is, the less information one can get from the ensemble^1^,^15^. Hence the quantumness of the two parts of a complete set of POPS must be “large” enough, at least larger than 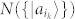 and 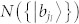, so that one can only get limited information and the states in the set cannot be locally distinguished. Therefore the local quantumness of an ensemble determines the local indistinguishability.

Theorem 2 gives a constructive distinguishing procedure to judge the local distinguishability for a complete set of POPS. Let {|*ψ_i_*〉 = |*a_i_*〉 ⊗ |*b_i_*〉} be a complete set of POPS, corresponding to two sets *ε^A^* = {|*a_i_*〉} and *ε^B^* = {|*b_i_*〉}. To verify the inequality (3), one needs to find all the subsets involved. This can be done in the following way.

- Decompose the sets *ε^A^* and *ε^B^* into subsets, 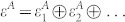, 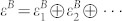 with the states in each subset constituting a single set. This process employs the corresponding local measurement 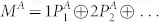 and 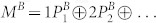, where 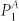, 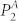, 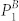, 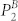, … is the projections to the spaces spanned by 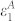, 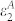, 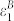, 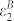, … respectively.
- Find the overlapped states between these subsets, 
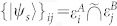
, where 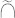 means to find the states with the same subscripts by classical communication. For example, if 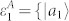, 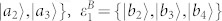, then 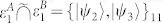. Each {|*ψ_s_*〉 = |*a_s_*〉 ⊗ |*b_s_*〉}*_ij_* corresponds to two new subsets 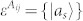 and 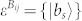.

We repeat the above process *n* rounds for the new subsets until each of those new subsets has only one element or both *A* and *B* parts cannot be decomposed further. At last we have subset {|*ψ_k_*〉 = |*a_k_*〉 ⊗ |*b_k_*〉}*_st_*_,…,*ij*_ (here *st*, …, *ij* are the measurement outcomes) corresponding to two new sets 
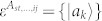
 and 
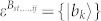
, where both 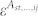 and 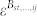 are single sets. If all the final single subsets have one element, the set {|*ψ_i_*〉} can be locally distinguished.

As an example, there is a complete set of POPS for 3 ⊗ 4 system, 
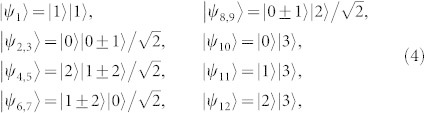
which corresponds to two sets, *ε^A^* = {|*a*_1_〉 = |1〉, |*a*_2,3_〉 = |0〉, |*a*_4,5_〉 = |2〉, 
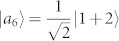
, 
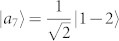
, 
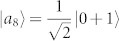
, 
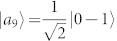
, |*a*_10_〉 = |0〉, |*a*_11_〉 = |1〉, |*a*_12_〉 = |2〉} and *ε^B^* = {|*b*_1_〉 = |1〉, 
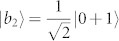
, 
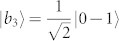
, 
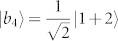
, 
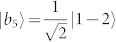
, |*b*_6,7_〉 = |0〉, |*b*_8,9_〉 = |2〉, |*b*_10,11,12_〉 = |3〉}. The distinguishing process is as follows.

Round 1: (i) There are 3 = dim(span *ε^A^*) linear independent states in *ε^A^* satisfying 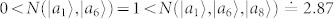. So *ε^A^* is a single set. Hence the first measurement should not be applied to *A* side. We denote 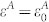 (here 0 means to do no measurement). For *B* side, we have decomposition 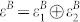, where 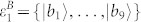 and 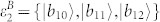 are all single sets. Hence 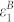 is a single set as there are 3 = dim(span 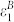) linear independent states satisfying 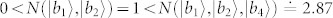. This decomposition employs measurement *M^B^* = 1(|0〉〈0| + |1〉〈1| + |2〉〈2|) + 2|3〉〈3| to distinguish 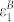 from 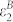.

(ii) Find the overlapped states by classical communication, 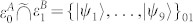, 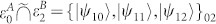. They correspond to four new sets 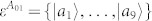, 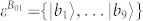, 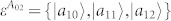, 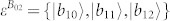.

Round 2: (i) Do decomposition for the above four new sets. Note 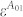, 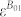 are all single sets (the states |*a*_4_〉, |*a*_6_〉, |*a*_8_〉 in 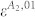 are linear independent and satisfy 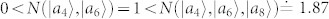, and 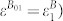). Therefore the set {|*ψ*_1_〉, …, |*ψ*_9_〉}_01_ is the subset described in Theorem 2. In fact they are the nine locally indistinguishable states in^9^. But we can distinguish the set {|*ψ*_10_〉, |*ψ*_11_〉, |*ψ*_12_〉}_02_ further since we have decomposition 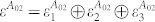, where 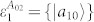, 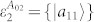, 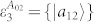. This employs measurement *M^A^* = 1|0〉〈0| + 2|1〉〈1| + 3|2〉〈2|. However, 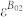 is single, denote 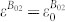.

(ii) Find overlapped states by classical communication, 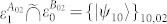, 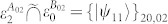, 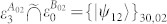. All the above new subsets either have one element each or both sides are single ones.

Finally we can divide the set (4) into four parts, {|*ψ*_1_〉, …, |*ψ*_9_〉}_01_, {|*ψ*_10_〉}_10,02_, {|*ψ*_11_〉}_20,02_, {|*ψ*_12_〉}_30,02_, and the set cannot be completely distinguished due to the subset {|*ψ*_1_〉, …, |*ψ*_9_〉}_01_.

### The quantumness of semi-classical states

**Figure 1:**
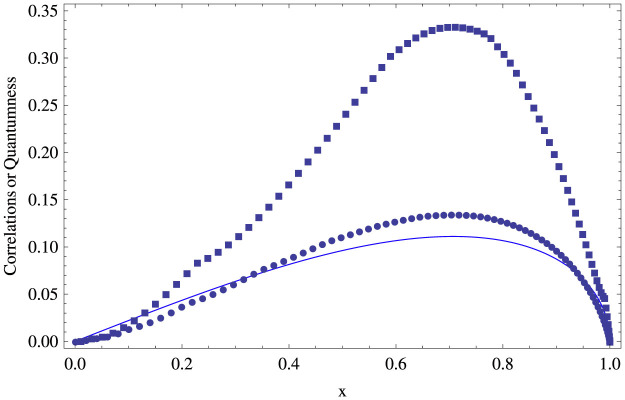
The comparison between the quantum correlations and the ensemble quantumness for a 3 ⊗ 3 quantum-classical state *ρ_x_* = 1/3(|0〉〈0| ⊗ |0〉〈0| + |1〉〈1| ⊗ |1〉〈1| + |*ϕ*_2_〉〈*ϕ*_2_| ⊗ |2〉〈2|), where |*ϕ*_2_〉 = cos *θ*|0〉 + sin *θ*|2〉 and horizontal ordinate is the overlap *x* = |〈0|*ϕ*_2_〉| = |cos *θ*|. Quantum correlations, quantum discord (circle doted line) and quantum deficit (square dotted line), and the ensemble quantumness, non-commutativity (solid line), for *ρ_x_* all get minimal at *x* = 0, 1 while get maximal at 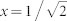.

The non-commutativity defined by (1) can be also used to describe the quantumness for bipartite semi-classical states. For a given semi-classical state, 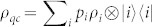, the corresponding ensemble *ε* = {*p_i_*, *ρ_i_*} is fixed (see Methods). Denote *X_i_* = *p_i_ρ_i_*. We have the non-commutativity describing the quantumness for the state *ρ_qc_*, 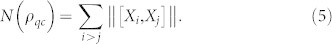
It is easy to check that *N*(*ρ_qc_*) satisfies the following properties, which actually makes it a candidate for quantum-correlation measures for semi-classical states^4^: (a) it is positive, (b) it is zero for classical-classical correlated states, (c) it is invariance under local-unitary transformations, and (d) it is non-increasing when an ancillary system is introduced. This quantity *N*(*ρ_qc_*) describes the quantumness of semi-classical states. It is different from those quantum correlation measures, such as quantum discord, quantum deficit, etc., which are based on the measurements and their estimations involve extremely complicated optimization process. Interestingly, while the quantity *N*(*ρ_qc_*) can be easily computed, it shows similar behavior to other quantum correlation measures for semi-classical states. Hence, instead of those quantum correlation measures, one can use the non-commutativity to characterize the quantum correlations of semi-classical states *ρ_qc_*. Moreover, non-commutativity provides a tool to explore the relation between the ensemble quantumness and those quantum correlation measures. Fig. 1 shows the comparison between quantum correlations and ensemble quantumness (non-commutativity) for a 3 ⊗ 3 system for which it is extremely difficult to calculate quantum correlations analytically.

## Discussion

We have proposed non-commutativity as a quantumness measure for an ensemble. It has been shown that the local ensemble quantumness, instead of quantum correlation measure, like quantum discord, quantum deficit, etc., which is a function of a density operator, accounts for the local indistinguishability for a complete set of POPS. It implies that the quantumness of local ensembles must satisfy certain conditions so that the states in the ensemble cannot be locally distinguished.

A constructive distinguishing procedure to obtain the concrete local measurements and classical communications has been presented to judge the local indistinguishability for a complete set of POPS. Our approach to judge the local indistinguishability can also be directly extended to distinguish a complete set of multipartite POPS or a non-complete set of POPS, when the local operations are restricted within nondestructive projective measurements.

Due to that one semi-classical state corresponds to one quantum ensemble, the non-commutativity has been shown to be able to characterize the ensemble quantumness for classical-quantum or quantum-classical systems.

## Methods

### Proof of the property (2) of the non-commutativity *N*

Consider two pure *n* dimensional states *A* = |*ϕ*〉〈*ϕ*| and *B* = |*ψ*〉〈*ψ*|. Denote 〈*ϕ*|*ψ*〉 = *xe^iθ^* with *x* ∈ [0, 1], *θ* ∈ [0, 2*π*). Then [*A*, *B*] = *xe^iθ^*|*ϕ*〉〈*ψ*| − *xe*^−*iθ*^|*ψ*〉〈*ϕ*|. Under the base {|*ϕ*〉 = |*ϕ*_0_〉, |*ϕ*_1_〉, …, |*ϕ_n_*_−1_〉}, one gets 
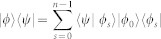
. [*A*, *B*] can then be expressed as 
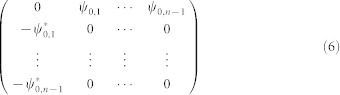
where *ψ_i_*_,*j*_ = 〈*ϕ_i_*|*ψ*〉〈*ψ*|*ϕ_j_*〉 and 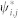 is the conjugate of *ψ_i_*_,*j*_. The eigenvalues of the above matrix are 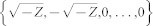, where 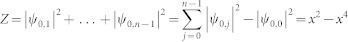. One gets 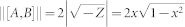. Therefore for two pure states |*ψ*〉 and |*ϕ*〉, ||[|*ϕ*〉, |*ψ*〉]|| is 0 when |〈*ϕ*|*ψ*〉| = 0 or 1, and 1 when 
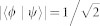
.

### Proof of Lemma 1

(a) ⇒ (b) First choose any vector in *ε*, say, 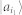. Then choose the second vector 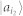 in *ε* such that it is not equal to or orthogonal to 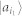, i.e., 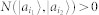. The existence of 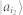 is guaranteed by the fact that *ε* is a single set. Next choose the third vector 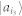 in *ε* such that it is independent of 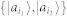, and also not orthogonal to 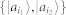, i.e., 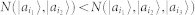. Or else, if 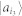 does not exist, then *ε* can be direct sum decomposed into two parts. Continuing with the above process, we can finally get *m* linear independent states 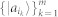 in *ε* satisfying inequality (2).

(b) ⇒ (c) A nondestructive projective measurement is described by an observable, *M*, an Hermitian operator such that all |*a_i_*〉 in *ε* are the eigenvectors of *M*. If 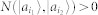, 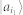 and 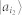 are neither identical nor orthogonal. Hence the corresponding eigenvalues of 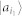 and 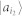 must be equal, since the eigenvectors corresponding to different eigenvalues are orthogonal. If 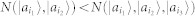, 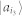 is at least not orthogonal to one of 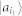 and 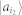. For instance, suppose 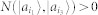, then the eigenvalues corresponding to 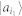 and 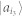 are equal. Therefore we have that if (2) is satisfied, those *m* linear independent 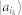 have the same eigenvalues of *M*. Therefore *M* becomes an identity (apart of real factor) and it cannot be used to distinguish the states in *ε*.

(c) ⇒ (a) Suppose the set is not a single one and has a decomposition *ε* = *ε*_1_ ⊕ *ε*_2_. Then one can use a corresponding nondestructive projective measurement *M* = *aI*_1_ ⊕ *bI*_2_, where *a* ≠ *b*, *I*_1_, *I*_2_ are identity operators, to distinguish the states in *ε*_1_ from *ε*_2_. Hence if *M* cannot do any in distinguishing the states in *ε*, *ε* must be a single set, that is, statement (a) holds.

### The unique expression of semi-classical states

Let 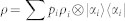 be an arbitrary given semi-classical state, where {|*α_i_*〉} is an orthogonal base. Suppose there exists another orthogonal base {|*β_i_*〉} such that *ρ* = *σ*, where 
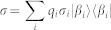
. Let 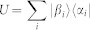 be the unitary operator such that *U*|*α_i_*〉 = |*β_i_*〉, (*U*)*_ij_* = *u_ij_* = 〈*α_i_*|*β_j_*〉, 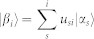. So *σ* can be reexpressed as 
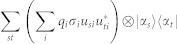
. Let 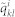 be the entries of *q_i_σ_i_*. Set 
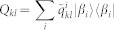
. Then *ρ* = *σ* implies that 
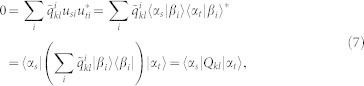
for *s* ≠ *t*, which means that both {|*α_i_*〉} and {|*β_i_*〉} are the eigenvectors of *Q_kl_*, and 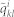 are the corresponding eigenvalues. Note that the set {*q_i_σ_i_*} of state *σ* can be divided into degenerate part (in which all the states are identical) and non-degenerate part (in which all the states are mutually different). For non-degenerate part, taking over all *k*, *l*, one will find that the intersection of the eigenspaces belonging to the eigenvalues 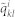 must be one dimensional, namely, |*α_i_*〉 = |*β_i_*〉 and then *p_i_ρ_i_* = *q_i_σ_i_*. For degenerate part, without loss of generality, assume *q*_1_*σ*_1_ = *q*_2_*σ*_2_, then *ρ* = *σ* means *p*_1_*ρ*_1_ ⊗ |*α*_1_〉〈*α*_1_| + *p*_2_*ρ*_2_ ⊗ |*α*_2_〉〈*α*_2_| = *q*_1_*σ*_1_ ⊗ (|*β*_1_〉〈*β*_1_| + |*β*_2_〉〈*β*_2_|). Therefore *p*_1_*ρ*_1_ = *p*_2_*ρ*_2_ = *q*_1_*σ*_1_ = *q*_2_*σ*_2_. Finally we get *p_i_ρ_i_* = *q_i_σ_i_*, ∀*i*.

## Author Contributions

T.M., M.Z., Y.W. and S.M. wrote the main manuscript text. All authors reviewed the manuscript.
